# Obituary.—Dr. Robert C. Hutchings

**Published:** 1894-02

**Authors:** 


### OBITUARY.

### DR. ROBERT C. HUTCHINGS

(20 Johnson Place, Buffalo, N. Y.)

Died at Colorado Springs, Colorado, January 18, 1894, of hæmorrhage of the lungs He had been suffering with consumption for the last three years. He spent the winter of 1891–92 at the Bermuda Islands; the winter of 1892–93 with his mother in the south of England, and this winter he went to Colorado Springs, where he died, as above stated. He was born in England, November 17, 1847; was educated in the schools of his native home. At the age of seventeen he joined the English Navy. Soon after retiring from the latter, he entered the Ontario Veterinary College as a student, and graduated from the same institution with honors in 1871. The first seven years of his professional life were spent at Watertown, N. Y. In 1878 he moved to Buffalo, N. Y., where he enjoyed a fine practice. He leaves a widow and daughter to mourn his loss.

